# Spontaneous spinal epidural hematoma management: a case series and literature review

**DOI:** 10.1038/scsandc.2016.43

**Published:** 2017-02-02

**Authors:** Kyle Raasck, Ahmed A Habis, Ahmed Aoude, Leonardo Simões, Fernando Barros, Rudy Reindl, Peter Jarzem

**Affiliations:** 1 Division of Orthopaedic Surgery, McGill University, Montreal, Quebec, Canada; 2 Instituto de Ortopedia e Traumatologia, Oeste D’Or Hospital, Campo Grande, Rio de Janeiro, Brazil

**Keywords:** Neurosurgery, Neurological manifestations, Prognosis, Neurological disorders, Outcomes research

## Abstract

**Objective:**

Spontaneous spinal epidural hematoma (SSEH) manifests from blood accumulating in the epidural space, compressing the spinal cord and leading to acute neurological deficits. Standard therapy is decompressive laminectomy, although spontaneous recoveries have been reported. Sub-optimal therapeutic principles contribute to SSEH’s 5.7% mortality—which patient will benefit from surgery remains unclear. This study aims to investigate parameters that affect SSEH’s progression, outlining a best-practice therapeutic approach.

**Materials and methods:**

Literature review yielded 65 cases from 12 studies. Furthermore, 6 cases were presented from our institution. All data were analyzed under American Spinal Injury Association (ASIA) score guidelines.

**Results:**

Fifty percent of SSEH patients do not fully recover. In all, 30% of patients who presented with an ASIA score of A did not improve with surgery, although every SSEH patient who presented at C or D improved. Spontaneous recovery is rare—only 23% of patients were treated conservatively. Seventy-three percent of those made a full recovery, as opposed to the 48% improvement in patients managed surgically. Thirty-three percent of patients managed conservatively had an initial score of A or B, all improving to a score of D or E without surgery. Regardless, conservative management tends toward low-risk presentations. Patients managed conservatively were three times as likely to have an initial score of D than their surgically managed counterparts.

**Discussion:**

The degree of pre-operative neural deficit is a major prognostic factor. Conservative management has proven effective, although feasible only if spontaneous recovery is manifested. Decompressive laminectomy should continue to remain readily available, given the inverse correlation between operative interval and recovery.

## Introduction

Spontaneous spinal epidural hematoma (SSEH) is a rare disease that can lead to the acute onset of severe neurological deficits,^1^ requiring early diagnostics and rapid treatment to reduce the likelihood and severity of sensorimotor deficiencies.^2^ First described by Jackson^3^ the condition manifests by an accumulation of blood in the vertebral epidural space^1^ that can compress the spinal cord or spinal nerve roots. The ‘spontaneous’ refers to the atraumatic etiology and contributing multifactorial factors such as hemophilia, neoplasms, arteriovenous malformation, hypertension, anticoagulants, straining, sneezing or lifting.^4,5^ In fact, idiopathic cases account for only 40–60% of all SSEHs.^6^ The most widely accepted hypothesis for the source of bleeding is the venous system, as spinal epidural veins are unprotected from changes in abdominal or thoracic pressure. An increase in these pressures would increase intravenous pressure, leading to the vessel’s rupture.^7^ This etiological theory is congruent with SSEH literature, as 54% of patients with SSEH report a straining-associated event during the initial attack.^8^ However, Beatty and Winston^9^ argue for an arterial source of hemorrhage, at least in the cervical region, stipulating that intrathecal pressure is higher than venous pressure, which precludes venous bleeding. Hemorrhage etiology has been contested for decades, highlighting a salient element of SSEH in that its etiology remains obscure, given the paucity of documented cases.^10^

SSEH’s atraumatic etiology makes it an ideal clinical model to study the reversibility of acute spinal cord compression symptoms due to ischemic reperfusion injury and identify the critical time frame until irreversible damage occurs. To define and substantiate these parameters, a detailed registration must be created on a patient by patient basis: medical history, time of onset, evolution of symptoms, extent of sensorimotor deficits, position and size of hematoma, operative interval, type of intervention, time to recovery and remaining deficits post recovery. The condition’s rarity hampers the collection of data, and this limitation is why details on which type of SSEH patient will benefit from decompressive surgery remain unclear.^11^ The purpose of this study is to improve our understanding of SSEH through a case series of patients treated at our institutions as well as a review of the current literature to evaluate various management approaches and ultimately determine the optimal therapeutic strategy.

## Patients and methods

**Figure 1:**
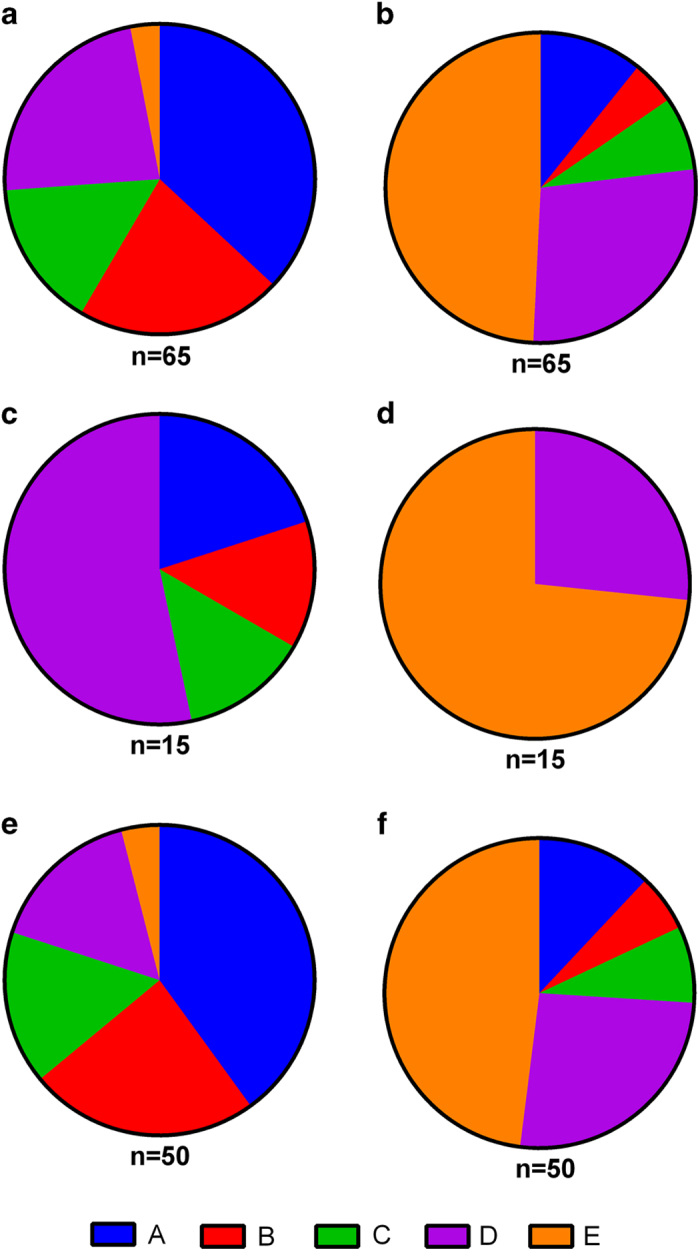
SSEH patient ASIA scores upon presentation (left column) and upon maximal resolution (right column). Out of 65 cases described (**a**, **b**), 15 were treated conservatively (**c**, **d**) and 50 were treated surgically (**e**, **f**). ASIA scores are indicated as colour.

SSEH pertinent peer reviewed publications featuring novel case studies from January 2000 to April 2015 were included in the analysis. The inclusion criteria for the literature review were as follows: (1) clinical studies on the management of SSEH and (2) detailed information for each case containing method of management and the neurological examination before and after treatment. From the 12 eligible studies based upon the above criteria, data from a total of 65 cases were extracted.^5,6,12–15^ If present, the following were collected: age, gender, type of intervention, operative interval, and neural deficit before and after treatment. If necessary, the degree of the patient’s neural deficit was converted into an American Spinal Injury Association (ASIA) Impairment Scale grade (AIS) so as to effectively compare and contrast. This has been summarized in Figure 1. Furthermore, all cases of SSEH treated conservatively or operatively at our institutions have been included in the following case series.

## Results

Literature review yielded a total of 65 SSEH patients, of which 15 were treated conservatively and 50 were treated surgically. Overall, mean age (+/- standard deviation) at the time of SSEH presentation was 53.2 (+/- 23) years. Of the 15 SSEH patients who were treated conservatively, 3 presented with an ASIA score of A (20%), 2 presented with a score of B (13%), 2 presented with a score of C (13%), and 8 presented with a score of D (53%). Upon maximal resolution of the SSEH, 4 resolved to an ASIA score of D (27%) and the remaining 11 fully recovered with a score of E (73%). Of the 50 SSEH patients, who were treated surgically, 20 presented with an ASIA score of A (40%), 12 presented with a score of B (24%), 8 presented with a score of C (16%), 8 presented a score of D (16%), and 2 presented with a score of E (4%). Upon maximal resolution of the SSEH, 6 resolved to an ASIA score of A (12%), 3 resolved to a score of B (6%), 4 resolved to a score of C (8%), 13 resolved to a score of D (26%) and the remaining 24 fully recovered with a score of E (48%).

## Statement of ethics

We certify that all applicable institutional and governmental regulations concerning the ethical use of human volunteers were followed during the course of this research.

## Case series

### Patient 1

**Figure 2:**
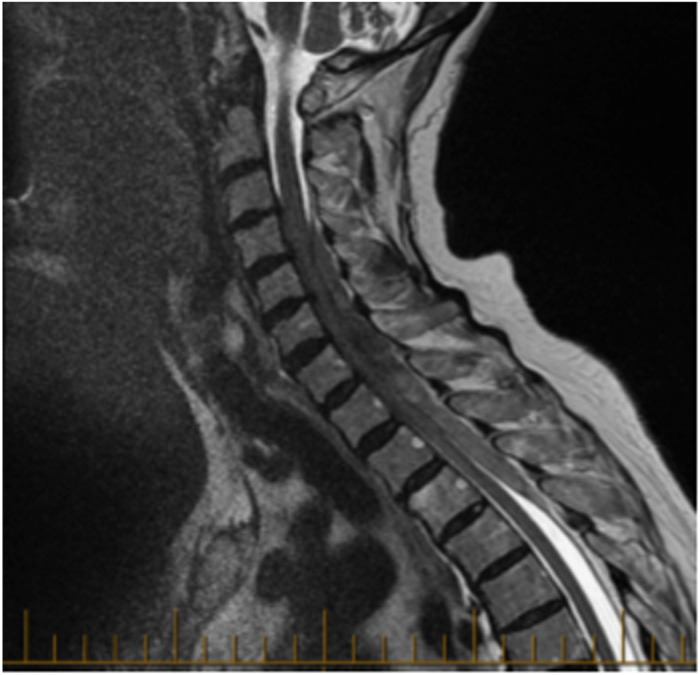
92-year-old female with hematoma from C4 to T4 measuring 1.2 -cm thick and 12.3 cm in length. Improved from ASIA C to ASIA D with surgery.

A 92-year-old female with a history of hypertension, angina, hypothyroidism and hypercholesterolemia presented with sudden interscapular pain followed by increasing weakness in her upper limbs that was more pronounced on her right side. On arrival to the hospital, neurological examination showed bilateral upper limb weakness with MRC score 3/5 on her right side and 4+/5 on her left. Her laboratory blood analyses were normal. Urgent magnetic resonance imaging (MRI) demonstrated an extensive eight-leveled epidural hematoma extending from C4 to T4, causing the spinal cord compression seen in Figure 2. The hematoma was 1.2 cm in thickness and 12.3 cm in length. There was no significant improvement in neurological status until the operation 21 h after the onset of her symptoms. She underwent decompressive laminectomy with evacuation of hematoma. Her ASIA score improved from C pre-operatively to D post-operatively. She has remained steady at an ASIA score of D upon presentation to her last follow-up 3 months after the surgery.

### Patient 2

**Figure 3:**
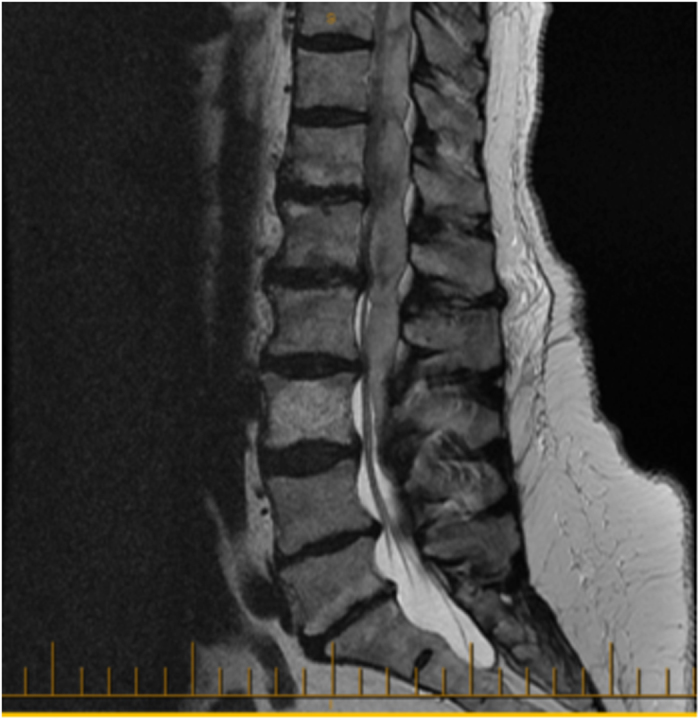
76-year-old female with hematoma from T7 to L1 measuring 1.2-cm thick and 15 cm in length. Improved from ASIA D to E with surgery.

A 76-year-old female with a history of hypertension, atrial fibrillation and anticoagulation therapy for deep vein thrombosis in her lower extremities presented with sudden and excruciating back pain. The pain rapidly progressed within a few hours, making her unable to walk. She also reported mild weakness and reduced sensation in her left lower limbs. Neurological examination revealed an MRC score 4-/5 weakness in her left leg while all other limbs were 5/5. Laboratory blood analyses were normal with an INR of 1.04. Urgent MRI showed a large ten-leveled epidural hematoma starting from T7 to L4, with a thickness of 1.2 cm and a length of 15 cm, causing the spinal cord compression seen in Figure 3. Laminectomy and hematoma evacuations were performed 28 h after the onset of her symptoms. The patient’s ASIA score was D pre-intervention and it did not change immediately following surgery. However, she fully recovered to an ASIA score of E at both her 6 and 12 week follow-ups.

### Patient 3

A 72-year-old male with a history of hypertension, atrial fibrillation and anticoagulation therapy (Cumarinic) presented with neck pain. The pain began rapidly and was followed by weakness in all limbs. On examination, he had weak hip flexion, hip adduction and knee extension. His laboratory blood analyses revealed an elevated INR of 3.2 but were otherwise normal. An urgent MRI scan identified a six-leveled epidural hematoma extending from C3 to T1 with a thickness of 0.9 cm and a length of 6.7 cm. Hematoma evacuation was performed 15 h after the onset of his symptoms. The patient’s ASIA score was C pre-operatively and immediately post-operatively. However, a follow-up at 3 months revealed an improvement to a score of D.

### Patient 4

**Figure 4:**
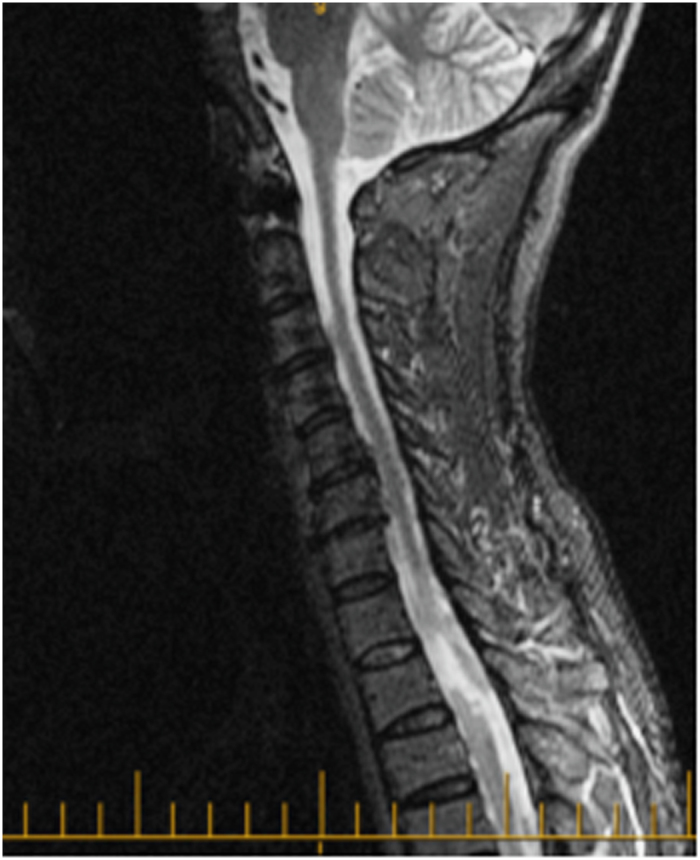
50-year-old female with hematoma from T1 to T5 measuring 0.65-cm thick and 8.9 cm in length. Improved from ASIA A to ASIA B with surgery.

A 50-year-old female was admitted for the treatment of fever and leukopenia. During her stay, she developed a complete motor deficit from T4 down. Her laboratory blood analyses revealed a low white cell count but were otherwise normal. An MRI done 3 days after the onset of her symptoms showed a four-leveled epidural hematoma extending from T1 to T5, causing the spinal cord compression seen in Figure 4. The hematoma was 0.65 cm in thickness and 8.9 cm in length. The patient underwent urgent decompressive laminectomy to evacuate the hematoma. Her ASIA score was A pre-operatively and did not change immediately post-operatively. However, a follow-up visit at 3 months showed improvement to an ASIA score of B.

### Patient 5

An 11-year-old female with no past medical history presented with severe neck pain. Within a few hours, she was unable to move her upper and lower limbs and the pain gradually worsened to an ASIA score of B. Her laboratory blood analyses were normal. Urgent computed tomography scan showed a six-leveled spinal epidural hematoma spanning from C3 to T1. It measured 0.5 cm in thickness and 4.2 cm in length. The initial plan was to surgically evacuate the hematoma. However, no surgical intervention was needed as the patient spontaneously recovered to an ASIA score of E.

### Patient 6

A 73-year-old male with a history of hypertension, atrial fibrillation and anticoagulation therapy (Cumarinic) presented with pneumonia. He was treated with antibiotics but deteriorated and was intubated for 2 days until his respiratory status improved. After extubation, he was noted to have complete upper and lower limbs’ paralysis. Laboratory blood analyses revealed an INR of 2.5. An urgent MRI showed a four-leveled epidural hematoma extending from C4 to C7. An urgent laminectomy and evacuation of hematoma was performed, although time between ictus and surgery can be estimated at >48 h. The patient’s ASIA score did not improve post-operatively, remaining at a score of A even at his 3-month follow-up.

## Discussion

### Etiology, epidemiology and pathophysiology

Despite the fact that SSEH accounts for <1% of all spine epidural lesions,^13^ with an estimated annual incidence of only 1 per million,^15^ the exceedingly high morbidity of untreated SSEH warrants its inclusion in the differential diagnosis of presentations suggesting spinal cord involvement.^2^ SSEH can present with features ranging from simple back pain with radiculopathy to complete paraplegia or quadriplegia, depending on the site and severity of spinal cord compression.^1^ The initial pain can mimic disc prolapse and is typically followed by progressive neurological deficits, the intensity of which are dependent on the severity of the bleeding.^16^ Any level of the spinal cord may be affected, but SSEH predominantly occurs in the posterior cervico-thoracic (C5–T2) and thoraco-lumbar (T10–L2) levels.^17,18^ The degree of neural deficit has a major prognostic effect, as recovery directly depends on the pre-operative neurological condition of the patient.^19^ Therefore, patients with minimal symptoms prior to therapy are more likely to completely recover than those with major deficits in sensorimotor functioning.^8,20,21^

The ASIA developed a set of standards that assess the degree and level of spinal cord injury to an individual, on scale of A—for most severe—to E—for normal. A hallmark indicator of neural function that has long been used in longitudinal studies, the ASIA score serves as an accurate marker for SSEH patients’ pre-operative functioning and subsequent prognosis.^22^ Therefore, a score should be immediately collected upon presentation.

Upon suspicion of SSEH, the immediate investigation of choice to confirm is MRI, supported by reports of the increased mean incidence of SSEH following the introduction of MRI into standard medical practice.^23^ This early recognition provides the opportunity for rapid, appropriate treatment, and can often lead to complete recovery with excellent neurological outcome.^2^ A computed tomography scan should be obtained if MRI is unavailable, as time is often a factor in the SSEH disease process.^24^ However, determining the appropriate treatment remains a cause for debate. Whether to remain conservative or intervene^4^ and, in the latter case, the time between ictus and decompressive surgery, are key factors that must be determined and refined, given the rapid neural deterioration SSEH can cause.^25^ Management principles remain sub-optimal, as supported by the disease-related mortality rate of 5.7%.^8^

The etiology of SSEH—more specifically the source of hemorrhage—has remained cause for debate for many a decade. The considerable body of evidence points to the venous system as the most likely source of hemorrhage, as the posterior epidural venous plexus does not contain valves and is largely unprotected from traumatic rupture.^7^ However, the hypothesis of an arterial source of bleeding has also been well supported.^9^ Given the sometimes rapid onset of SSEH, greater arterial flow is indicative of an arterial source, as opposed to the lesser flow generated by the venous system where intrathecal pressure is higher than venous pressure. In the present case series, we observed an equal amount of patients with rapid and slow onset of hematoma and the accompanying pain and neurological deficits. For example, patients 1, 2 and 3 reported sudden and severe symptoms, whereas patients 4, 5 and 6 exhibited a more gradual onset. This observation does not favor either etiological theory. However, it could illustrate an underlying and overlooked question: why must one source of bleeding preclude another? Perhaps an SSEH patient is just as likely to bleed from an artery as they are from a vein. Furthermore, although this arterial versus venous distinction may be of great academic interest, the source of bleeding is not a major prognostic factor once the epidural space has been infiltrated with blood. Detection of SSEH is limited to patient presentation typically occurring after the onset of symptoms, which is dependent on the presence of a hematoma. Therefore, the time between ictus and detection would remain independent from the source of bleeding, rendering the latter’s clinical significance obsolete.

What is of greater clinical importance is how easily the patient in question can develop the spontaneous bleeding in the epidural space. Oral anticoagulant use is rapidly becoming an identifiable risk factor for SSEH. Dziedzic *et al*.^12^ recently conducted a case series and reported oral anticoagulant use by five out of ten SSEH patients. Furthermore, in the present case study, four patients out of six were taking oral anticoagulants—two received Warfarin prescribed for atrial fibrillation, whereas another two patients used daily Aspirin. This treatment regimen could increase the likelihood of hemorrhage. Furthermore, in 54% of patients a straining-associated event preceded the initial attack.^8^ Therefore, anticoagulation therapy must be considered closely in SSEH patients.

### Management principles

At present, there is no consensus on the best-practice paradigm in SSEH treatment. Admittedly, this is easier said than done as SSEH’s decidedly cloudy etiology, low incidence and widely varying symptoms, which range from local vertebral pain to paresis, create a spectrum too wide for generalized treatment. In our case series, the patient age ranged from 11 to 92, whereas ASIA scores upon presentation ranged from A to D. The review of current literature revealed a similar trend, as shown in Figure 1. This marked variability has prompted the division of therapeutic strategies into two categories: surgical and conservative.

However, these management principles remain sub-optimal with a disease-related mortality of 5.7%. Furthermore, the literature review reveals that over 50% of patients continue to have some level of sensorimotor deficiency—that is, an ASIA score of A through D post-treatment (Figure 1b). This morbidity is alarmingly high and necessitates the need for improvement in the therapeutic approach. To this effect, the treatment options available, as well as their degree of success with the varying patient presentations pooled from over 12 studies, including the present case study, are discussed.

#### Surgical treatment

Decompressive laminectomy and hematoma evacuation are the standard surgical procedures upon diagnosis of SSEH. Two salient factors have been shown to have major prognostic implications—the degree of pre-operative neural deficit and the time interval between ictus and surgery.^2^

A hallmark indicator of pre-therapy neural deficit is the ASIA score. Across the 12 studies, it was observed that 30% of patients who presented with an ASIA score of A did not improve with surgery (Figures 1d and e). However, every SSEH patient who presented at an ASIA score of C or D improved with surgery. This is in agreement with our case series, as one of the two patients who presented with an ASIA score of A showed no improvement post surgery, although delay to intervention was present, while those with pre-operative ASIA C and D all improved. This validates the necessity to collect an ASIA score immediately upon presentation of SSEH-like symptoms, as it aids in developing a prognosis and determining the time and type of intervention needed. In fact, Liao *et al*.^26^ developed a protocol at Chang Gung Memorial Hospital that bases the time and type of intervention solely on the patient’s pre-operative ASIA score.

An emphasis has been placed on establishing an acceptable surgical time frame between ictus and intervention. In a series of 14 patients, Shin *et al*.^21^ described that patients who were operated on <12 h after the onset of symptoms scored significantly higher (84%) on the recovery scale set by the Japanese Orthopaedic Association than those who underwent surgery between 12 and 24 h after (64%) and those who were operated on 24h post symptom presentation (47%). Furthermore, in a recent meta-analysis, Bakker found the duration of paralysis pre-operatively to be the only independent predictor of poor outcome.^11^ This inverse relationship between time and outcome is congruent with our case series, as the only patient who showed no improvement was operated on over 48 h after the onset of the symptoms.

However, it is important to note that this particular patient’s pre-operative ASIA score was A, which also contributed to the lack of improvement post surgery. Another patient in our case series, who presented with an ASIA score of D, was only operated 28 h after surgery and still made a full recovery. Therefore, one could argue that SSEH can, when causing a large pre-operative neural deficit of ASIA score A or B, require priority emergency care. In contrast, when the neural deficit is of ASIA score C or D, urgent care is recommended but time is not as strong a factor.

A sliding scale of acceptable time intervals between ictus and surgery that is dependent on the patient’s pre-operative ASIA score could prove to be a useful prognostic and therapeutic tool. Liao *et al.*^27^ proposed a version of such an algorithm in 2009, where any signs of progressive deterioration with pre-operative ASIA score of B through D or an ASIA score of A lasting more than 12 h required an ‘emergent’ surgery. A pre-operative ASIA score of A lasting less than 12 h would be deemed ‘urgent’ and improving cases with a pre-operative ASIA of D were recommended to be solely observed. However, a more extensive patient registration which details the time of surgical interval, degree of initial deficit and outcome must be created to define statistically significant, quantifiable time frames for these various presentations of SSEH.

#### Conservative therapy

An increasing number of SSEH cases have been reportedly managed conservatively. In Bakker’s recent meta-analysis, they excluded all patients treated conservatively in each respective study they evaluated—this is understandable, as the factors precipitating its practice are poorly understood.^11^ Regardless, 23% of patients in the reviewed literature were managed conservatively as well as one out of the six in the present series.

The sudden increase in both the size of hematoma and neural deficit are possible during the conservative treatment. Cases initially treated conservatively have been reported to deteriorate even after a marked period of recovery, ultimately requiring surgery.^6^ Furthermore, increasing time between ictus and surgery, as established before, has a major negative prognostic effect. Therefore, conservative management should never be considered if working in a center without surgical spine services and imaging on hand.

Having established this, conservative management has proven to also be a successful management strategy in certain SSEH scenarios. In a study focusing on conservative management, Groen^28 ^ reported 84% of patients treated non-operatively recovered completely. However, the report was biased as the majority of the patients had a minor pre-operative neural deficit and small lesions. According to the literature review, patients managed conservatively were more than 3 times as likely to have an initial ASIA score of D—the least severe score before full recovery—than their surgically managed counterparts (Figures 1c and e). Interestingly, 26% of patients with an ASIA score of D after conservative observation were not operated on—perhaps, when taking into account the inherent risks for nerve damage, leaks and infections associated with a laminectomy, the risk-benefit was not favorable for surgical intervention (Figure 1e). Regardless, 73% of patients managed conservatively made a full recovery (ASIA score E) as opposed to the 48% who were managed surgically (Figures 1e and f). It must be emphasized that patients selected for conservative management had, on average, a smaller neural deficit than those who were managed surgically, and within this comparison lies an inherent bias.

However, it is of great curiosity that 20% of patients managed conservatively had an initial ASIA score of A, and an additional 13% had an initial score of B, all of whom improved without surgery to an ASIA score of D or E. Essentially, this signifies that patients have gone from complete paralysis below the site of injury to a complete recovery of function without surgical intervention. This spontaneous recovery in some SSEH patients is due to the gradual spread of hematoma throughout the epidural space, thereby decompressing the spinal cord and decreasing the neural deficit.^29^ It must be stipulated that this only happens in rare cases, and patients with large initial neural deficits should be managed surgically unless significant spontaneous recovery is observed as they are being prepared for surgery. This was the case with Patient 5, an 11-year-old female, in our case series. During preparation, the patient gradually recovered with a marked improvement in her upper and lower limbs strength. Her ASIA score improved from an initial B at first physical examination to a final ASIA score of E.

This phenomenon is an ideal model to atraumatically study the reversibility of acute spinal cord compression symptoms without intervention, although cases are so stark and, due to their complicated etiology, often ignored in studies. By continuing the collection of conservatively managed cases, perhaps we can identify and substantiate more parameters that indicate the likelihood of spontaneous recovery or degree of benefit from decompressive surgery. At present, non-operative treatment can be a reasonable option in a neurosurgical unit for patients presenting with minimal neural deficits, or in those where spontaneous recovery has started before or during surgical preparations. Observation should include repeat MRIs, and surgical intervention may still be necessary even after a period of marked recovery.

The purpose of this study and the accompanying literature review was to present our cases series with the aim to outline a best-practice therapeutic approach to treat the rare and debilitating condition that is SSEH. The etiology has yet to be precisely determined, although oral anticoagulants contribute as a risk factor. Prognosis is mostly determined by ASIA score upon presentation, although complete recovery from an initial ASIA score of A was observed. While there is strong support for the earliest possible decompressive surgery, the conservative approach remains a feasible option if spontaneous recovery is manifested. It is imperative to note, however, that decompressive laminectomy should be readily available should the patient’s neurological status begin to worsen or not improve shortly after presentation. The importance of surgical availability cannot be overemphasized, as the inverse correlation between the time elapsed between ictus and the surgery and the resulting extent of recovery has been extensively documented.

In brief, we recommend that close neurological monitoring and early imaging be completed for any patient with suspected SSEH, especially in those with anticoagulation therapy. Once confirmed on imaging, rapid determination of those requiring surgical intervention should be based on the evolution of their neurological status from time of symptom onset to the latest neurological examination by a trained spine surgeon. Therefore, any patient without sign of improvement or any patient with deterioration in ASIA score should be emergently treated by surgery. More case series including both surgical and conservative treatment are required to identify and substantiate more parameters that will further refine the optimal therapeutic solution proposed.
